# Recent Advances in Digital Biosensing Technology

**DOI:** 10.3390/bios12090673

**Published:** 2022-08-23

**Authors:** Kathrine Curtin, Bethany J. Fike, Brandi Binkley, Toktam Godary, Peng Li

**Affiliations:** 1 Department of Mechanical and Aerospace Engineering, West Virginia University, Morgantown, WV 26506, USA; 2 C. Eugene Bennett Department of Chemistry, West Virginia University, Morgantown, WV 26506, USA

**Keywords:** digital biosensing, digital ELISA, digital PCR, microfluidics, biosensor

## Abstract

Digital biosensing assays demonstrate remarkable advantages over conventional biosensing systems because of their ability to achieve single-molecule detection and absolute quantification. Unlike traditional low-abundance biomarking screening, digital-based biosensing systems reduce sample volumes significantly to the fL-nL level, which vastly reduces overall reagent consumption, improves reaction time and throughput, and enables high sensitivity and single target detection. This review presents the current technology for compartmentalizing reactions and their applications in detecting proteins and nucleic acids. We also analyze existing challenges and future opportunities associated with digital biosensing and research opportunities for developing integrated digital biosensing systems.

## 1. Introduction

Detection and analysis of proteins and nucleic acids from human fluids are essential for disease and environmental monitoring, public health, and clinical diagnostics. Because these molecules impact individuals’ and society’s health and safety, it is paramount that they are detected rapidly and accurately. PCR and ELISA are considered the “gold standards” for nucleic acid and protein detection, respectively. However, these techniques suffer from several hurdles that limit their broad application, especially at the point-of-care (POC). Many valuable biomarkers, such as cardiac troponin and HIV RNA, exist in very low concentrations. Biomarkers at the attomolar and femtomolar levels can be challenging to detect using bulk PCR or ELISA. When the concentration of target biomolecules is low, the corresponding detection signal may decrease to a level that cannot be distinguished from the background due to excessive dilution in bulk solutions [1]. In addition, low-level signals are more prone to the matrix effects making quantification results unreliable.

One strategy to overcome the issues in bulk biosensing method is to use digital biosensing. Digital biosensing is the process of compartmentalizing a bioassay reaction into a small fluid volume that either contains 0 or 1 target. Conventionally, PCR, ELISA, and other sensing assays are conducted on a bulk or ensemble scale and quantified based on the amplitude of the detectors’ response. In digital biosensing, the assay readout is based on the number of positive reaction vessels with a volume as low as the fL or pL [2]. Compared to conventional reaction bulk assays, digital biosensing offers several advantages. First, each biosensing reaction occurs in a small volume vessel. By significantly decreasing the reaction volume, the reaction kinetics and mass transfer is improved, while excessive dilution of signals is reduced [1,3]. Therefore, detecting single molecules in each reaction vessel becomes feasible. Second, digital biosensing enables absolute and digital quantification. Absolute quantification does not require known standards or controls, so the target of interest is directly quantified from the number of positive or negative reactions with high precision [1]. Since the quantification does not rely on the absolute signals, it provides better tolerance to the matrix effects or other factors that affects the biosensing reaction [1,4]. To date, digital biosensing has significantly improved detection limits for immunoassays and nucleic acid tests to the single molecule scale. Achieving this level of sensitivity enables early detection of valuable biomarkers.

Bioassay development and materials science advancements, including microdevice design, fabrication, and accessibility, have enabled digital biosensing to flourish over the past decade. Successfully translating conventional biosensing technology to droplet biosensing for nucleic acid and protein detection requires careful consideration of the biological processes and technical requirements for the assays. Digital biosensing assays must be wisely designed based on the target biomolecule of interest as the biological requirements and reaction steps differ across targets. These considerations influence design engineering, such as the optimal droplet generation method and the types of downstream fluid manipulation needed. These factors critically influence how the assay should be engineered and the needs of the assay for it to be successful. Our review aims to provide insight into the relationship between the technical requirements needed to measure different classes of biomolecule targets successfully.

In this review, we analyze the current state of digital biosensing technology for protein and nucleic acids. For each application, we review the latest technology and innovations for generating, manipulating, and sensing droplets, emphasizing overall system design, functionality, and assay sensitivity. Our analysis details the individual technical requirements and interdisciplinary design considerations for droplet protein and nucleic acids. Lastly, we discuss future directions and research opportunities in droplet biosensing technology.

## 2. Digital ELISA (dELISA)

Since its inception in 1971 by Engvall and Perlman [5], ELISA has become the prevailing standard for detecting and quantifying proteins, peptides, antibodies, and hormones from complex samples such as whole blood, blood plasma, and urine. Monitoring these biomolecules has become indispensable for diagnosing and monitoring disease and health. While many variants of ELISA have been developed, they all operate using the same basic principles, including: 1. well-plate immobilization of antigen either by direct or indirect methods; 2. plate blocking to reduce nonspecific binding; 3. detection using detection antibodies; 4. washing; 5. readout via a signal generation mechanism. Typically, ELISA is carried out using a well plate platform, and the quantification is achieved by establishing a calibration curve using known concentrations of target. ELISA can achieve a sensitivity at the level of low pg/mL, and offers high specificity, especially with sandwich assays. However, to further improve the sensitivity of conventional ELISA is extremely difficult due to the excessive dilution of signaling molecules in bulk solution. In addition, this method is subject to high interference levels from background signals and biological matrix, even with multiple washing steps.

In 2010, Rissin et al. [6] reported the first dELISA method achieving a detection sensitivity as low as 14 fg/mL for prostate-specific antigen (PSA) in serum samples. Instead of detecting the signals from bulk ELISA reactions, this method employed magnetic beads as the solid support for immune recognition and compartmentalized each individual bead into microwells for signal generation and detection. Thus, the quantification was achieved by counting the number of positive microwells without the need of a calibration curve. Since then, dELISA technology has made considerable progress in improving detection sensitivity, throughput, and operation. In this section, we discuss recent digital technology that enables high-sensitivity protein detection with an emphasis on device engineering.

### 2.1. Technical Needs for dELISA

dELISA typically involve the multiple steps included in conventional ELISA, including incubation, washing, and detection. For dELISA to be effective, several technical requirements must be considered. Conventional ELISAs use 96-well plates to physically anchor the immunocomplex for washing and detection. Because of this requirement, dELISA assays must also contain a solid support for immunocomplex formation. Nanoparticles and magnetic beads are among the most common materials for dELISA since they are commercially available and have high surface area to volume ratios. Magnetic beads are typically micron or nano-sized spheres of iron oxide covered with a polymeric material and can be controlled by an external magnet [7].

The ELISA protocol is generally translated to a digital form by forming an immunocomplex-bead and partitioning the beads into individual reactions. Once partitioned, either the substrate is added, or the substrate can be added with the immunocomplex-bead in the first step. During compartmentalization, the solid support must be effectively transferred into the droplet or partition. Several strategies have been employed to partition the reaction for protein detection, including microstructure arrays, microfluidics, and magnetic interactions to meet the needs of dELISA. There must be efficient reagents and solid support encapsulation during compartmentalization, careful volume control, sufficient incubation and signal detection time, and downstream fluid manipulation if needed. For POC applications, it is essential to design dELISA technology that shortens incubation time, limits the number of washing steps, and allows simple data acquisition and interpretation. After the ELISA reaction is complete, several readout signals are available to realize the result, including fluorescence, electrical, chemiluminescence, and SERS (surface-enhanced Raman scattering).

### 2.2. Methods and Technology for Compartmentalizing the Solid Supports in dELISA

#### 2.2.1. Droplet Microarrays

Droplet microarrays are a common strategy for droplet ELISA because they enable detection with high throughput without complex droplet generation systems. The droplet microarrays are typically an array of hydrophobic regions surrounded by hydrophilic borders [8,9]. With droplet microarrays, there are two main ways to generate droplets: on-array or off-array. On-array microdroplet arrays are generated by using a flow cell to pass the liquid over the hydrophobic-hydrophilic surface. As the liquid passes over the surface, the droplets will remain in the hydrophilic region [10]. Microdroplet arrays can also be generated using droplet stamping where the droplets are stamped into the hydrophilic region using a microstructure. Once the droplets are formed, they can undergo the ELISA process using magnetic bead capture and immunocomplex formation [11]. An early work demonstrated a typical sandwich ELISA using inkjet printing to generate droplets for the reaction [12]. Off-array generation of droplets involves making them and transporting them to the chip using a pipette or another transfer method [8]. For example, a one-pot immunoassay was developed to detect IL-8 using immunocapture and proximity ligation assay. The droplets were produced using a vortex method and the resulting fluorescence signals were read out after transfer to an imaging chip. This design maintained high sensitivity at 0.793 pM [13].

#### 2.2.2. Microwell Arrays

Microwell arrays are another type of array that compartmentalizes reactions. Instead of using hydrophobic and hydrophilic interactions like in droplet microarrays, microwells use physical structures to separate them. The use of wells offers many advantages with biosensing as wells hold a fixed volume of liquid, making quantification simple due to the consistent volume of every well. In addition, there is a physical boundary between each portion, which reduces the likelihood of cross-contamination between wells. Although the use of wells offers these benefits, the method is limited by the number of available wells within the array. Additionally, the wells must be composed of a biocompatible material to prevent reaction fouling and interference.

**Figure 1:**
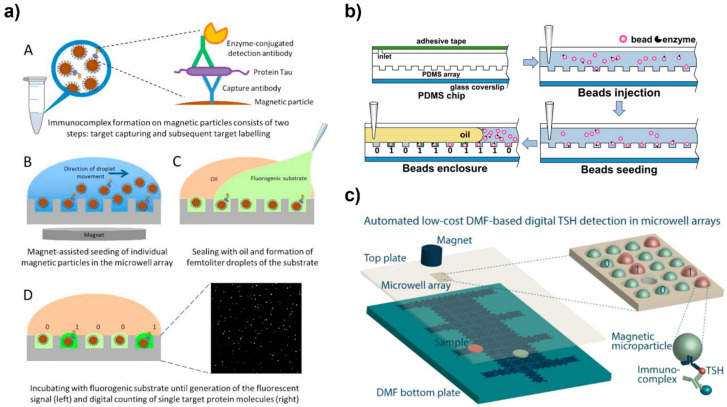
(**a**) Schematic of microwell array workflow including loading and oil sealing using a pipette and fluorescence readout. Reprinted with permission Analytical Chimica Acta Volume 1015, Perez-Ruis et al. “Digital ELISA for the quantification of attomolar concentrations of Alzheimer’s disease biomarker protein Tau in biological samples” pp. 74–81 (accessed on 27 July 2022) Copyright 2018 with permission from Elsevier [16]. (**b**) Microwell array loading using a flow cell. Reprinted with permission Biosensors and Bioelectronics Volume 139, Sun et al. “Power-free polymethylsiloxane femtoliter-sized arrays for bead-based digital immunoassays” p. 111339 (accessed on 28 July 2022) Copyright 2019 with permission from Elsevier [17]. (**c**) Microwell array seeding using magnetic and DMF. Reprinted with permission from Leirs, K.; Dal Dosso, F.; Perez-Ruiz, E.; Decrop, D.; Cops, R.; Huff, J.; Hayden, M.; Collier, N.; Yu, K.X.Z.; Brown, S.; Lammertyn, J. Bridging the Gap between Digital Assays and Point-of-Care Testing: Automated, Low Cost, and Ultrasensitive Detection of Thyroid Stimulating Hormone. *Anal. Chem.* **2022**. https://doi.org/10.1021/acs.analchem.2c00480 (accessed on 25 July 2022). Copyright 2022 American Chemical Society [18].

Magnetic beads primarily serve as the solid support structure for the immunocomplex because they can be manipulated with an external magnet. The beads are easily loaded and anchored into the microwells during washing steps using a magnetic force. With this design, the magnetic bead-immunocomplex is either formed before loading into the wells or after loading. For example, for detection of PSA using a microwell array, the PSA was incubated with the capture antibody-magnetic beads and detection antibody. The beads were then washed and mixed with the substrate [14,15]. After complex formation, the beads were loaded into the microwell array using a micropipette and flow cell. After the beads settled in the wells using a magnet, the fluorescence signal was detected. Alternatively, the substrate can be added directly to the microwells as a separate step [16]. In this case, magnetic beads form the immunocomplex and are washed. They are then loaded into the microwells without the addition of substrate. Then, the substrate is added directly to the microwells. In both cases, oil is used to seal the wells and prevent evaporation. These designs offer flexibility in the amount of loading steps and workflow (Figure 1).

In microwell arrays, reaction solutions are loaded into the microwells using a flow cell, digital microfluidics (DMF), centrifugation, or microfluidic channels. Manual loading with a pipette is among the simplest design for microwell seeding. With a pipette, the bead solution is added to the chip and then the oil and substate are added to seal the reaction (Figure 1a) [16]. In flow cell loading, the desired liquid is pumped into the cell and the wells are loaded with the liquid (Figure 1b). Then, the wells are sealed using oil and airflow [19]. Vacuum pressure can also be used to draw the bead solution over the microwells [20]. Centrifugation is an automated option for loading microwell arrays. These lab-on-a-disc devices are loaded sequentially with the magnetic bead solution and oil sealant, so when centrifuged, the wells are loaded and sealed [21,22].

DMF coupled with magnetic beads is another automated option for loading microwell arrays. By applying a magnet beneath the microwell array, a magnetic bead suspension is moved across the array and the bead fall into the array because of the magnetic force [8]. This technique improves the loading efficiency to 98% with a CV (coefficient of variation) of 0.9% to achieve 10 aM to 90 fM detection. This work demonstrated the feasibility of their approach to “print” an array that is loaded with magnetic beads. In the work of Leirs et al. [18], DMF with magnetic force was used to seed the microwells as an alternative to flow cell loading. This design is more automated than using a flow cell and allowed for high seeding efficiencies of 97.6% (Figure 1c).

Some microwell array platforms have achieved advanced control with microfluidics [2]. Song incorporated a microwell biosensor into a microfluidic chip for a pre-equilibrium enzyme-linked immunosorbent assay (PEdELISA) system to detect cancer biomarkers [23]. The PEdELISA consisted of only two steps: one for loading and mixing to form the magnetic bead-immuno complex and one for labeling with HRP (horseradish peroxidase) for readout. The ultrafast and automated microfluidic system enabled fast detection with femtomolar selectivity in less than 10 min. The design is based on capturing the “pre-equilibrium” result so that incubation times could be significantly shortened. Combining microwell arrays with microfluidics offers more control of reagent loading for multistep reactions like ELISA [24]. Microfluidic loading with valve control has also been shown to improve microwell loading speed with automation. Wang et al. [25] developed a system that integrated a microwell assay chamber and on-chip pumps that can be programmed for fluidic delivery to the sandwich immunoassay. In this design, the microwells were surface modified with the capture antibody for immunocomplex formation. Then, the microfluidic channels passed the subsequent reaction fluids over the microwells for ELISA. 

Kan et al. [9] demonstrated a way to increase the loading efficiency of beads into microwells by using magnetic forces and meniscus sweeping. To enhance the loading of the beads, the air plug-aqueous droplets-air plug droplet chain were dragged across the microwell array in a back-and-forth motion. The capillary and magnetic forces both caused the magnetic beads to fall into the respective wells. By improving bead loading, attomolar level sensitivity was achieved.

#### 2.2.3. Microstructure Arrays

Microcapillary arrays have also been used for protein analysis [26]. The microcapillary strategy is like that of a microwell in that the single targets can be captured in individual reaction zones. A reporter microcapillary array that contained millions of high aspect ratio microcapillaries allowed for high surface area binding to detect proteins using a magnetic bead immunocomplex. In this design, the microcapillaries were loaded with a micropipette.

Like microcapillaries, nanopillars have been coupled with dELISA. In a study by Li et al. [27] antibody-conjugated pillars were used to provide an excess of immunocomplex binding sites for cytokine detection. They studied the influence of the pillar’s dimensions on capture and detection. The cross-sectional area was critical as it is the space where cytokine binding and labeling with SERS tags occurs. Upon optimization, the nanopillars achieved attomolar level detection sensitivity. Nanopillars are also advantageous for microdevices because they aid in maintaining the tension between two immiscible liquids when using moving parts. A microfluidic platform that utilized micropillars for protein detection allowed careful actuation of oil and water droplets [28]. The micropillars prevented fouling of the oil and water interface for robust movement of magnetic detect beads through the device chambers. In a work by Uddin et al. [29], micropillars were coupled with droplet microfluidics to miniaturize a conventional ELISA. These micropillars reduced assay time to 15 min and achieved a sensitivity of <10 pg/mL for cardiac troponin I.

#### 2.2.4. Microfluidics

**Figure 2:**
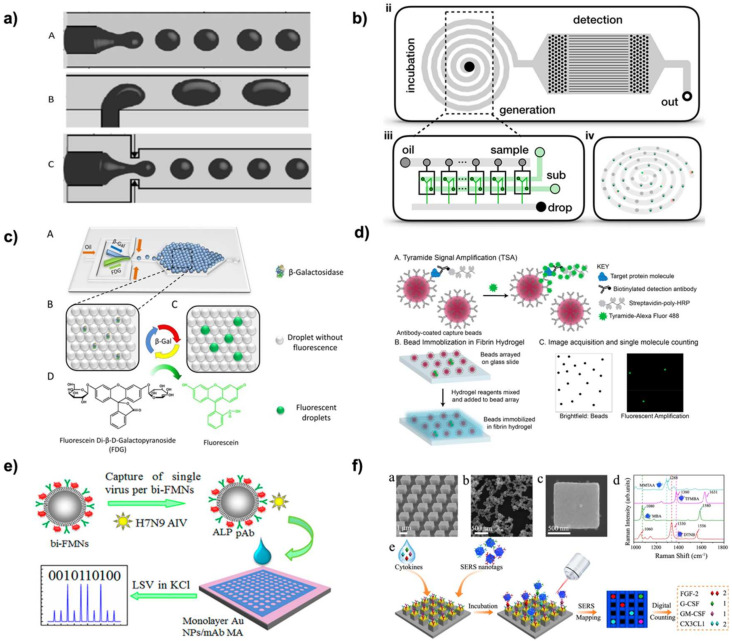
(**a**) Schematics of most common droplet formation methods: Co-Flow (**A**), T-Junction (**B**), and Flow-Focusing (**C**). Reproduced with permission from Shang, L.; Cheng, Y.; Zhao, Y. Emerging Droplet Microfluidics. *Chem. Rev.* **2017**, *117*, 7964–8040. https://doi.org/10.1021/acs.chemrev.6b00848 (accessed on 15 August 2022) [3]. (**b**) Parallel droplet generation device for exosome dELISA. Reprinted with permission from Yang, Z.; Atiyas, Y.; Shen, H.; Siedlik, M.J.; Wu, J.; Beard, K.; Fonar, G.; Dolle, J.P.; Smith, D.H.; Eberwine, J.H.; Meaney, D.F.; Issadore, D.A. Ultrasensitive Single Extracellular Vesicle Detection Using High Throughput Droplet Digital Enzyme-Linked Immunosorbent Assay. *Nano Lett.* **2022**. https://doi.org/10.1021/acs.nanolett.2c00274 (accessed on 22 July 2022). Copyright 2022 American Chemical Society [33]. (**c**) Flow focusing microfluidic device with reaction chamber. Reprinted from Guan, Z.; Zou, Y.; Zhang, M.; Lv, J.; Shen, H.; Yang, P.; Zhang, H.; Zhu, Z.; James Yang, C. A Highly Parallel Microfluidic Droplet Method Enabling Single-Molecule Counting for Digital Enzyme Detection. *Biomicrofluidics* **2014**, *8*, 014110. https://doi.org/10.1063/1.4866766 (accessed on 27 July 2022). AIP Publishing [34]. (**d**) Bead immobilization in hydrogel for microstructure free dELISA. Reprinted with permission from Maley, A.M.; Garden, P.M.; Walt, D.R. Simplified Digital Enzyme-Linked Immunosorbent Assay Using Tyramide Signal Amplification and Fibrin Hydrogels. *ACS Sens.* **2020**, *5*, 3037–3042. https://doi.org/10.1021/acssensors.0c01661 (accessed on 20 July 2022). Copyright 2020 American Chemical Society [35]. (**e**) Electrochemical dELISA schematic. Reprinted with permission from Wu, Z.; Guo, W.-J.; Bai, Y.-Y.; Zhang, L.; Hu, J.; Pang, D.-W.; Zhang, Z.-L. Digital Single Virus Electrochemical Enzyme-Linked Immunoassay for Ultrasensitive H7N9 Avian Influenza Virus Counting. *Anal. Chem.* **2018**, *90*, 1683–1690. https://doi.org/10.1021/acs.analchem.7b03281 (accessed on 22 July 2022). Copyright 2017 American Chemical Society [36]. (**f**) SERS digital nanochip SEM, schematic, and counting for cytokine detection Reproduced with permission from Nature Communications under http://creativecommons.org/licenses/by/4.0/ (accessed on 27 July 2022) [27].

Microfluidic devices are one of the most common platforms for droplet-based dELISA because droplets can be easily generated and manipulated within the device for downstream processing and readout. Droplet generation in microfluidics is typically done using flow-focusing, co-flow, or T-junctions. (Figure 2a) [2,4] Flow focusing, developed in 2002 by Anna et al. [30], involves a cross junction where two oil phases are perpendicular to a single channel of the aqueous liquid. These three streams meet at a single point and the difference in channel dimensions and flow rates causes the oil phase to pinch the aqueous phase into droplets. Co-Flow droplet formation, developed in 2004 by Cramer et al. [31], is a method where the inlet for the liquid forming droplets (aqueous phase) is inserted into the surrounding liquid (oil phase). The injection system is typically upright for this method, so gravity will help form droplets. T-Junction droplet formation, first reported by Thorsen et al., in 2001 [32], has the two liquid inlets perpendicular to each other. The oil phase flows straight along the channel, and the aqueous phase inlet is perpendicular to the channel’s flow. When the aqueous droplets reach a specific size, the oil pinches the droplet closed due to the shear stress from the flow of the liquids. All these methods require precise control of the flow rates, determined with the use of syringe pumps, of both liquids to obtain the correct size droplet needed for the analysis [4]. In addition, these methods make the prediction of droplet size difficult because they can change based on a small change in the flow rate as well as the size of the channel being used. These methods are called “passive” methods of droplet generation because there is no other assistance in forming the droplets. Additionally, device material must be compatible with the droplet generation and biosensing. To date, a wide range of materials have been reported for droplet microfluidics, including poly(dimethyl) siloxane (PDMS), glass, silicon, thermoplastics, and 3D printing resin. PDMS is the most commonly used polymers for digital biosensing because it is inexpensive and well-established in microfabrication processes [3]. However, it has drawbacks in terms of stability. To ensure stable droplet formation, the choice of channel material must be compatible with the continuous phase. For example, generating water-in-oil droplet prefers the channel surface to be hydrophobic. Alternatively, the channel surface can be chemically modified to control the surface property. For example, salinization is often performed to enable generating water-in-oil droplets in glass microdevices.

Alternatively, “active” methods are utilized and have additional assistance from an external source. These external sources have four main categories (electrical, magnetic, thermal, and mechanical), utilizing a key trait in the liquid-forming droplets. For example, using a magnetic field would increase the capability of droplet formation when using a ferrofluid as the dispersed phase [37]. Another method to generate and control droplets is by using pneumatic valves [2]. These microvalves can be turned on or off to control the liquid flow and cause the formation of droplets and the mixing of different reagents [29,38]. In addition, a combination of these stated methods may be used in complex droplet formation, such as in forming multiple-emulsion droplets or with multiple component droplets. In addition, the formed droplets can be manipulated in various ways, such as sorting, merging, splitting, mixing, and trapping. The available range of droplet generation and manipulation methods allows for droplets to be used for a variety of applications, especially for biosensing [3].

Several designs have been presented to improve droplet generation throughput. Improving droplet generation throughput is critical for refining assay sensitivity and achieving highly parallel reactions. A droplet microfluidic was designed to achieve a high throughput of 20 million droplets/min, which is more than 100 times greater than other microfluidic devices (Figure 2b) [33]. This device generated droplets in parallel using multiple droplet generation zones, which then converged into the reaction and detection zones on the chips. The level of throughput on this device enabled ultrasensitive detection of extracellular vesicles. Another microfluidic droplet platform was used to generate droplets in a highly parallel manner to produce 10^7^ droplets within 10 min [39]. This device was further integrated with smartphone technology to visualize the results easily.

In microfluidic devices for dELISA, the ELISA assay can be conducted by using microfluidic methods to encapsulate the bead-immunocomplex for digital readout. Microfluidic-based digitization separates the bulk bead-based ELISA reaction into microscale droplets. This typical magnetic bead procedure involves the capture antibody conjugation on the magnetic beads, antigen capture, and then incubation with an enzyme-labeled detection antibody. The magnetic beads are then washed and added to the microfluidic device so they can be merged with droplets containing the enzyme substrate for detection. The droplets are then incubated and readout using either fluorescence or SERS methods under continuous flow [33,40,41] or within an incubation chamber (Figure 2c) [34,42,43,44].

Alternatively, the ELISA steps can be simplified on a microfluidic device using droplet generation and manipulation. Magnetic tweezers and magnets are especially useful techniques in this case. These tools utilize magnetic fields to manipulate and hold the magnetic beads in places for good extraction efficiency, flexible movement control of magnetic beads, and efficient washing steps [45]. For extraction, the magnetic tweezers extract the magnetic beads from the sample droplet and the magnet keeps them in place, and a washing droplet is used to disperse and wash the beads [46]. Washing is done by capturing the droplet containing the magnetic beads with a magnet and merging it with washing droplets [47]. The magnet will hold the beads in place for merging with the next droplet or reaction step. This strategy has also been used to concentrate the antigen in a specific zone for detection. Sensitivity and washing steps can be significantly improved to the pg level by concentrating the beads [48,49,50]. The entire movement of the magnetic droplet relies on the mass of the beads, the magnet itself, and the surface properties of the device. During washing steps, a magnet can also be used to rotate and shake the beads to increase washing efficiency [28]. It has been found that even though a magnet can hold the beads in place, high flow rates can still wash away the beads, which makes it essential to optimize your magnetic bead process for ELISA [51]. For example, a continuous flow microfluidic device generated droplets containing the sample and capture antibody-magnetic beads [52]. The droplets were then merged with the fluorescent-labeled detection antibody. Magnetic tweezers were used to capture the magnetic beads and an upstream washing buffer was used to wash away any excess detection antibody. In the capture zone, a fluorescence detector captured the resulting signal.

### 2.3. Readout Methods for dELISA

The readout method of an assay plays a vital role in the final output of droplet-based ELISA. Many readout methods for dELISA have been reported, including fluorescence, electrical, and surface enhanced Raman scattering (SERS). It should be noted that although colorimetric detection is one of the simplest and most common signals for conventional ELILSA, it is often not preferred for dELISA due to decreased optical lengths in small reaction vessels and the need of detecting both the bead signal and the reaction signal. Herein, we focus on recent developments in readout methods for dELISA and an extensive summary of recent works for dELISA is summarized in Table 1.

#### 2.3.1. Fluorescence

Fluorescence is the most used signal readout for dELISA due to its high sensitivity in small volumes and high multiplexing potential. A number of fluorescence techniques have been applied for dELISA including tyramide signal amplification, enzymatic reactions, and fluorescently labeled beads. Signals can be detected using fluorescence microscopy/imaging system, smartphone imaging, and flow cytometry for dELISA. Simplifying readout methods are highly desirable to meet the requirements for developing POC testing. A structure-free microarray was developed as another method for digitizing fluorescence ELISA. Capture beads were embedded in a layer of fibrin hydrogel instead of using physical barriers as discussed above, which simplified the dELISA assay procedures (Figure 2d) [35]. The hydrogel layer prevents bead movement during each reaction step and is a fast and simple way to compartmentalize the reaction. The tyramide fluorescence signal amplification system was reduced from five steps to three steps by combining the protein capture and antibody labeling step and by using a tyramide–fluorophore conjugate. This device achieved a limit of detection (LOD) of 1 fM for IL-6.

A smartphone is an attractive detector for POC testing with many advantages such as widely availability, having user-friendly tools, and being cost-effective. A smartphone device was coupled with a megascale droplet generation system to produce and measure up to 10^6^ droplets/second [38]. The high throughput droplet generation system used 120 parallel focusing channels. The smartphone was equipped with a LED excitation system that uses a modulated light sequence so that individual droplets could be resolved by the camera. This mobile platform was applied for the simultaneous detection of GM-CSF and IL-6. For multiplexed measurement, fluorescently labeled beads were labeled with their respective antibody. Upon antigen capture and signal generation, the smartphone device excites both the fluorescence bead and substrate. The software algorithm then differentiates the signals for each target bead and the fluorescence substrate. In this way, the signal generated from the enzyme could be associated with the respective fluorescence bead. The device achieved an LOD of 0.0045 pg/mL and 0.0070 pg/mL, respectively. Similarly, this encoded bead design was also used in a microwell array for multiplexed detection of IL-4, IL-6, and IFN-γ [53]. In this design, a microwell array on a disc was seeded with encoded beads. Instead of using a smartphone device, the microwells were imaged with fluorescence microscopy. This device achieved LODs of IL-4, IL-6, and IFN-γ as 0.082 pg/mL 0.097 pg/mL, and 0.079 pg/mL, respectively.

In addition to using the intensity of fluorescence signals, fluorescence has also been used for tracking the movement of particles as a means of counting positive reactions. Akama et al. [54] used magnetic nanoparticles with 550 nm diameter as a substrate of the immunocomplex and a label for signal detection. The notable feature of their method is that it does not require any signal amplification, lasers, filter, or expensive camera. For this purpose, each magnetic nanoparticle motion is analyzed under bright-field and fluorescence microscopy. Three movement classifications have been reported depending on their diffusion behavior: free diffusion, tethered diffusion, and nonspecific attachment. Free diffusion is related to the magnetic particle that only has a capture antibody on its surface. In contrast, tethered diffusion shows the particle’s motion with a complete sandwiched immunocomplex with the target antigen. The magnetic nanoparticle with an attachment of capture antibody to detection antibody, without any target antigen, is related with the nonspecific attachment classification. They reported that digital HoNon-ELISA has a 9-fold higher sensitivity than traditional ELISA and a 1.7-fold lower sensitivity than digital ELISA. Later, the system was further optimized to achieve multiplexed digital HoNon-ELISA. For PSA detection, LODs of 9.3 × 10^−2^, 5.9 × 10^−2^, and 5.5 × 10^−2^ pg/mL were reported for digital HoNon-ELISA, multiplexed digital HoNon-ELISA, and digital ELISA, respectively, which indicates that this method has the potential to reach similar sensitivity to standard dELISA.

Fluorescence signals can also be detected using flow cytometry for high throughput analysis. For instance, Akama et al. [55] introduced a droplet-free digital ELISA that utilizes a conventional flow cytometer for digital counting. They used the Horseradish peroxidase (HRP) enzyme labeled with an immunocomplex on top of magnetic beads. In addition, a tyramide signal amplification system was employed to concentrate the enzymatic reaction product to distinguish targeted beads during the flow cytometry. With the presence of hydrogen peroxide, horseradish peroxidase can convert tyramide into a radical intermediate, which forms a covalent bond with aromatic compounds on a protein surface. Tyramide was prelabeled with biotin or a fluorescent dye to track the signal from a single target molecule under flow cytometry. They reported 95% efficiency for a single-bead detection via flow cytometry. Compared with the conventional ELISA, this method offers a 20-fold higher sensitivity with an improved limit of detection at 0.09 mlU/mL for Hepatitis B.

#### 2.3.2. Electrical

Electrical impedance and electrochemical reactions have also been used in dELISA reactions. These methods use simple electronics that can be easily confined to a handheld device [56]. An electrical impedance device was designed with a capture antibody functionalized surface. After binding, any unbound beads were washed away. Brightfield images were taken of the bound beads for counting. Then, the detection beads were eluted from the chamber and passed through the electrical impedance sensors where the change in resistance correlated to the number of beads. By counting the changes in resistance, the amount of IL-6 was quantified from the sample. The device achieved an LOD of 50 pM.

Another dELISA assay used electrochemical methods to measure H7N9 influenza virus [36]. Capture antibody alkaline phosphatase (ALP) magnetic nanospheres were used to capture the viruses. By controlling the concentration of beds, it was ensured that a single virus would be captured by one bead. The complex was then loaded to a detection antibody functionalized microelectrode array. Here, the ALP catalyzes the dephosphorylation the substrate which reduces and deposits metal ions onto the electrode surface (Figure 2e). Linear sweep voltammetry (LSV) is used to generated signals that are counted as “0” or “1” based on digital analysis. The device measured a low detection limit of 7.8 fg/mL.

#### 2.3.3. Surface Enhanced Raman Scattering (SERS)

The traditional colorimetric assays have low sensitivity and are limited to organic dyes that exhibit low extinction coefficients. In contrast, metal nanoparticles with various morphologies display much better extinction coefficients. Gold and silver nanoparticles are the most prevalent among all metal nanoparticles since they have been showing thousands of times better results than organic dyes in the visible region. Localized surface plasmon resonance (LSPR) is the main reason for their unique optical properties, which comes from the oscillation of free electrons on the surface of metal nanoparticles under light excitation [57]. SERS is a fingerprint analytical technique that uses this feature of metal nanoparticles to enhance Raman signals. Integrating SERS with droplet microfluidics showed successful outputs for monitoring ELISA [48,58,59].

Alternatively, Li et al. [27] studied the SERS barcode readout with a digital nanopillar platform (Figure 2f). They designed this system for multiplex quantifying a single cytokine molecule via four targets. Multiple SERS nanotags for simultaneous detection has been shown to be a promising multiplexing strategy. After antigen capture, the target-specific SERS tag labeled detection antibody binds, causing a signature response. The key factor of this method was activating SERS nanotags by single particles for confocal SERS mapping. Their SERS nanotag was based on Au–Ag alloy nanobox, showing a better enhancement factor than spherical gold nanoparticles and pure silver nanobox. Each type of these four antigens had a particular nanotag to generate a unique Raman signal. Thus, each of the four SERS nanotag included one Raman reporter coupled with its corresponding specific detection antibody on its surface. Lastly, the SERS nanotag signal’s presence or absence on the nanopillar platform was presented as a percentage of active pillars. Then, this value is applied for total cytokine quantification. With the power of the narrow line width of Raman spectra, this technique enabled detection of multiple cytokines sensitively and simultaneously. A similar nanostructure design was used to compartmentalize a sandwich reaction for the detection of dopamine [59]. The 3D nanopillars compartmentalized the signal reactions for SERS digital readout. The assay measured to 1 pM of dopamine.

**Table 1:** Summary of existing dELISA methods.

Target	Platform	Generation Method	Readout	Limit of Detection	Range	Time	Reference
IL-8	Microdroplet Array	Vortex system	Fluorescence	0.793 pM buffer 1.54 pM whole blood	0–300 pg/mL	N/A	[13]
Aβ42 peptide	Microdroplet array	Flow cell	Fluorescence	N/A	N/A	N/A	[10]
Influenza A	Microdroplet Array	Droplet SETS	Fluorescence	0.032 hemagglutination units/reaction	N/A	40 min	[11]
IgA	Microdroplet Array	Inkjet printing	Fluorescence	N/A	6 ng/mL to 50 ng/mL	N/A	[12]
Cytokines	Nanopillar	Manual loading	SERS	0.044 ng/mL	N/A	30 min	[27]
cardiac troponin I (cTnI)	Micropillar array	Pumping	Fluorescence	9.75 pg/mL	0 to 1500 pg/mL	10–15 min	[29]
amyloid B	Micropillar Array	Manual loading with magnetic actuation	Fluorescence	10 pg/mL	12.5–200 pg/mL	45 min	[28]
SARS-CoV-2-IL6	Microwell Array	Programmed microfluidic loading	Fluorescence	0.4 pg/mL	sub-pg/mL to ng/mL	9 min	[60]
PSA	Microwell Array	Microfluidic loading	Fluorescence	0.093 pg/mL	0.1 pg/mL to 200 pg/L	1.5 h	[54]
PSA and bhCG	Microwell Array	Manual loading	Bright field imaging	0.060 pg mL^−1^, 2.84 pg mL^−1^	N/A	N/A	[61]
TNF-α	Microwell Array	Microfluidic loading	Fluorescence	3.0 aM	Max concentration 240 fM	N/A	[25]
Influenza A	Microwell Array	Hydrophobic-hydrophilic interaction	Fluorescence	4 ± 1 fM	0 to 100 fM	~2 h	[15]
TSH	Microwell Array	EWOD	Fluorescence	0.0013 μIU/mL	N/A	N/A	[18]
Tau	Microwell Array	Magnetic actuation	Fluorescence	24 ± 7 aM	1 × 10^−16^ to 1 × 10^13^	~20 min	[16]
β-galactosidase	Microwell array	Microfluidic loading	Fluorescence	100 fM	N/A	N/A	[19]
TNF-a, IL-6, IL-1a, and IL-1b	Microwell Array	Microfluidic loading	Fluorescence	21 fg/mL, 3 fg/mL, 5 fg/mL, 43 fg/mL	N/A	N/A	[20]
IL-4, IL-6, and IFN-γ.	Microwell Array	Microfluidic loading	Fluorescence	0.183 pg, 0.175 pg, 0.084 pg	N/A	N/A	[53]
Prostate-specific antigen (PSA)	Microwell Array	Flow Cell	Nanoparticle Enhanced	0.059 pg/mL	N/A	N/A	[62]
Interleukin 6 (IL6)	Microwell Array	Flow Cell	Nanoparticle Enhanced	0.039 pg/mL	N/A	N/A	[62]
PSA	Microwell array on a disk	Centrifugal Force	Fluorescence	10 zM and 2 aM	0.011 pg/mL up to 100 pg/mL	N/A	[22]
β-galactosidase and PSA	Microwell Array	Flow Cell	Fluorescence	2.0 aM and 10 zM	10 zM to 1 fM and 0 to 200 aM	10 min to 5 h	[14]
proteins—PSA and tumor necrosis factor-α (TNF-α)—to	Microwell Array	Centrifugal Force	Fluorescence	50 aM and 150 aM	N/A	N/A	[6]
PSA	Microwell Array	Centrifugal Force	Fluorescence	0.008 pg/mL	8 fg/mL to 100 pg/mL	N/A	[63]
alpha-fetoprotein (AFP)	Microwell Array	Microfluidic loading	Fluorescence	1 fg/mL	1 to 100 fg/mL	N/A	[64]
β-galactosidase and TNF-α	Microwell Array	Flow Cell	Fluorescence	930 zM and 50.48 fg/mL	1 aM to 1 fM	30 min	[17]
Cytokine	Microwell Array	Microfluidic loading	Fluorescence	N/A	10^4^	30 min	[23]
IL17a, 1L12p70, p24, interferon alpha	Microwell Array	Microfluidic loading	Fluorescence	0.7 aM 0.092 aM, 9.1 aM 45.9 aM	N/A	N/A	[9]
β-galactosidase and alkaline phosphatase (ALP)	Microwell Array	Microfluidic loading	Fluorescence	N/A	N/A	N/A	[19]
IGF-1R	Microwell Array	Microfluidic loading	Fluorescence	0.011 pg/mL and 0.016 pg/mL	10 fg/mL to 1 ng/mL	N/A	[24]
Up to 16 targets	Microwell Array	Centrifugal force	Fluorescence	0.07 IU mL^−1^	fg/mL to pg/mL	<1.5 h	[21]
IgA	Microwell Array	Inkjet dispensing	Fluorescence	N/A	0 to 50 ng/mL	3 min	[65]
IL-6	Microfluidic	Microwell	Electrical impedance	21.8 aM	six orders or magnitude	~1 h	[56]
H7N9 virus	Microarray	Manual loading	Ectrochemical	of 7.8 fg/mL	0.01 to 1.5 pg/mL	1 h	[36]
IL-6	Microarray	Bead immobilization	Fluorescence	1 fM	0.1 fM to 100 fM	N/A	[35]
HBsAg	Magnetic Beads	Droplet free	Fluorescence	0.09 mIU/mL	4 orders of magnitude	~1 h	[55]
IL-6 and HBsAg	Magnetic Beads	Droplet free	Fluorescence	0.1 pg/mL and 0.013 IU/mL	N/A	N/A	[66]
GM-CSF and IL6	Microfluidic	Parallel droplets generator	Fluorescence	0.0045 pg/mL (320 aM) and 0.0070 pg/mL (350 aM)	0–8 pg/mL	10 min	[39]
Prostate-specific antigen (PSA)	Microfluidic	Flow focusing	SERS tags	<0.1 ng/mL	0.05 to 200 ng/mL	174 droplets per minute	[50]
Dual of free-PSA and total-PSA	Microfluidic	T-junction	SERS tags	<0.1 ng/mL	0.05 to 100 ng/mL	10 min	[58]
SARS-CoV-2	Microfluidic	T-junction	SERS tags	0.22 PFU/mL	0 to 100 PFU/mL	≤10 min	[41]
Zika virus NS1	Microfluidic	Parallel flow focusing generation	Fluorescence	62.5 ng/mL	N/A	~9 min	[67]
Vesicles	Microfluidic	Parallel droplets generator	Fluorescence	9 EVs/μL	2 orders of magnitude	5 min	[33]
SARS-CoV-2 RNA	Microfluidic	T junction	SERS	0.22 PFU/mL	log 0.8 to 2	10 min	[41]
IL-6 and mTOR	Microfluidic	T-junction	Fluorescence	25 pmol/L and 800 pmol/L	N/A	Incubation time down to 27 min	[52]
IL-10	Microfluidic	Flow focusing	Fluorescence	0.14 pg/mL	N/A	N/A	[43]
teatnus protein	Microfluidic	Flow focusing	Fluorescence	0.1 IU/mL	0.1 IU/mL to 1 IU/mL	~30 min	[44]
β-galactosidase	Microfluidic	Flow focusing	Fluorescence	N/A	N/A	4 h	[34]
IL-6	Microfluidic	Flow focusing	Fluorescence	6 pg/mL	10 pg/mL to 2 ng/mL	N/A	[68]
IFNγ and IL-2	Microfluidic	Flow focusing	Fluorescence	30 aM and 20 aM	0 to 100 fM	N/A	[69]
TSH	Microfluidic	Injection	Fluorescence	40 pM	5 miIU/L	N/A	[45]
AFP and β-galactosidase	Microfluidic	Flow focusing	Fluorescence	N/A	5 fM to 250 fM	~3 h	[42]
glucose, LDH, bile acids	Microfluidic	Flow focusing	Fluorescence	70 μM	N/A	N/A	[38]

Legend: surface-enhanced Raman scattering (SERS).

To date, there are a range of strategies available for designing dELISA including the compartmentalization platform and readout. These design considerations aid in improving assay performance in terms of limit of detection and dynamic range. 

## 3. Digital Biosensing for Nucleic Acids

Nucleic acids are another important class of biomarkers. To achieve high sensitivity detection of nucleic acids, Polymerase Chain Reaction (PCR) is often employed to amplify the target sequences [70]. PCR has allowed for significant advancements in medical diagnostics as a tool for amplifying and detecting nucleic acids from biological samples. The technique is highly sensitive and can produce millions of copies of genetic material that can be used for sequencing, cloning, and analysis. Utilizing genetic information to diagnose and monitor disease has proven to be incredibly valuable and continues to serve healthcare fields [71]. Traditionally, PCR has been performed as a bulk reaction with a single result. In bulk reactions, the measurement is taken in one of two ways: real-time detection and endpoint detection. With real-time detection, the number of copies in the solution is determined based on the number of cycles it takes for the fluorescence intensity to pass a given threshold. It can also be used for qualitative detection where only the intensity over a particular threshold is needed. With endpoint detection, measurements are taken after amplification has been completed, typically with gel or capillary electrophoresis [72,73]. Both detection methods produce reasonable results. However, the bulk reaction could be affected significantly by inhibitors or bias in amplification, which leads to errors in quantification. The bulk reaction also offers many challenges due to the need for complex instrumentation as well as the length of time required for amplification. As an alternative and more accurate method to quantify nucleic acids, digital readout has been developed. Digital PCR (dPCR) first emerged in 1992 [12], though it was not formally named until 1999 [70], when Vogelstein and Kinzler reported a PCR approach that converted the analog result of PCR signals to a linear digital signal. With digital PCR, the bulk reaction is divided into small portions to capture individual copies of the target. Each portion would contain either “1” or “0” of the target, which shows positive and negative, respectively. Each portion is analyzed individually, and the quantity of positive and negative portions can be used to determine the absolute quantity of the beginning sample [70]. Since then, there has been much effort into using digital technology for nucleic acid sensing applications. Technology that simplifies the workflow into compact and automated systems are highly desirable as this allows for more POC applications.

In addition to PCR, many isothermal amplification techniques can also be converted to a digital detection platform, including Loop-Mediated Isothermal Amplification (LAMP) [74], Recombinase Polymerase Amplification (RPA) [75], Nucleic Acid Sequence-Based Amplification (NASBA) [76], Rolling Circle Amplification (RCA) [77], Strand Displacement Amplification (SDA) [78], and Helicase-Dependent Amplification (HDA) [79], among others. Isothermal amplification has benefits over PCR because these methods allow amplification in a short amount of time. Additionally, due to being held at a constant temperature instead of the thermocycling process of PCR, the complexity of the instruments necessary is reduced.

### 3.1. Technical Needs for Digital PCR

Key steps in performing dPCR include reaction mixture preparation, compartmentalization, amplification, and detection. Compared to dELISA, dPCR does not involve a series of incubation and washing steps and require additional solid supports for the reaction, which allows simpler fluid control systems. Using small volumes enhances the efficiency of the reaction by reducing the reaction time and potential inhibition and enabling single molecule capture. However, the challenging part for dPCR is the amplification reaction in small volumes. Extra care must be taken to establish a stable chemical environment, maintain the sample integrity, and reduce evaporation. There are some reports of using temperature-phase-dependent gels to keep the droplets stable. In one instance, an agarose solution is used to prepare the PCR mixture at room temperature, where the agarose is liquid. The liquid phase is emulsified in oil to form droplets, which undergo PCR amplification. During amplification, the droplets remain stable, and after thermocycling, the droplets are cooled to 4 °C, at which point the agarose forms solid gel beads. These beads can be removed from the oil phase for individual analysis [71].

Most of the amplification experiments require heating and controlling the temperature. Depending on the temperature range, there are a variety of heating methods available. The most common heating method used in commercial thermocyclers is thermoelectric heating. This method utilizes an effect known as the Peltier effect, where when two different metals are joined, and electric current pass through, one metal heats while the other cools [80]. Other possible means of heating the sample, including Joule heating [81], Surface Acoustic Wave (SAW) heating [82], Photonic heating [83], induction heating [84], microwave heating [85], solar heating [86], and body temperature heating [87] have all been demonstrated with promising results.

In addition, sample preparation steps including cell lysis, extraction, and concentration are necessary. These processing steps are done prior to compartmentalization; however, integrating sample preparation into an “all-in-one” assay is preferred, especially for POC applications.

The final necessity for nucleic acid tests in the detection and readout methods. Many bulk amplification methods use real-time detection, while endpoint detection is used with digital methods. With both types of detection, there are two common methods of monitoring the results with fluorescence detection: a photosensor and a camera. With a photosensor, a system of lasers and mirrors along with a photomultiplier tube (PMT) is set up. The laser travels to the channel and individual droplets are detected. The signal then travels through the system and the PMT detects the signal and converts the readings into a graph for visual analysis. With the camera-based system, a charge-coupled device (CCD) or complementary metal-oxide-semiconductor (CMOS) based system the fluorophores are excited, then the area is scanned with the camera, and finally, the image is shown and can be used for analysis [88]. Alternative methods of detection in bulk PCR reactions include colorimetric signal, electrochemical signal, electrophoresis, and surface-enhanced Raman scattering (SERS) [89].

### 3.2. Methods and Technology for Compartmentalizing dPCR

Many compartmentalization methods discussed for dELISA such as microwell array and droplet microfluidics have also been widely used for dPCR applications. Here, we briefly describe the general compartmentalization strategies and focus on techniques that are specifically allowed by dPCR due to its simple assay requirement. An extensive summary of recent works for digital nucleic acid detection is summarized in Table 2.

#### 3.2.1. Microwell and Microdroplet Arrays

**Figure 3:**
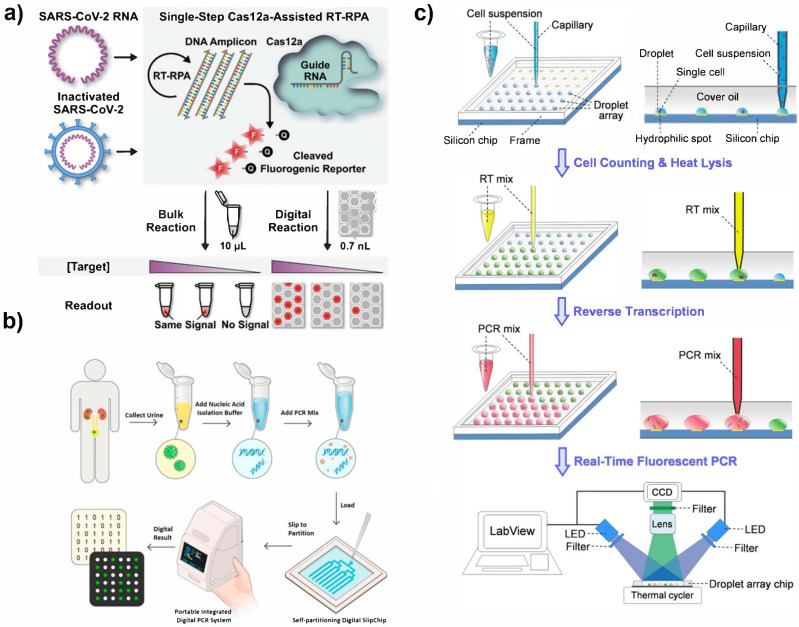
(**a**) CRISPR microwell array workflow. Reprinted with permission from Advanced Science [90] under Creative Commons https://creativecommons.org/licenses/by/4.0/ (accessed on 25 July 2022). (**b**) Workflow of SlipChip device for digital PCR. Reprinted from Biosensors and Bioelectronics, Volume 175, Xu et al., “Portable integrated digital PCR system from the point-of-care quantification of BK virus from urine samples” p. 112908, Copyright 2021 with permission from Elsevier (accessed on 26 July 2022) [100]. (**c**) Capillary probe droplet printing for RT-PCR. Reproduced with permission from Scientific Reports under Creative Commons http://creativecommons.org/licenses/by/4.0/ (accessed on 27 July 2022) [98].

Microwell arrays have been applied for digital nucleic acid detection because of their simplicity and ability to control reaction volumes (Figure 3a) [90]. Like with ELISA, microwell arrays for nucleic acid detection can be loaded using a range of techniques. Flow cell loading [91,92], vacuum-assisted loading and oil-driven digitization [93,94,95,96], pressure loading [97], dispensing robots [98,99], and self-induced partitioning via SlipChip [100,101,102] have all been used for loading microwell arrays for nucleic acid applications.

One microwell-based method is with the use of a SlipChip (Figure 3b) [100]. With a SlipChip, two sets of microwells fit together to form a single well for the reaction to occur. The reagents for the bulk reaction are divided so that each type of well only holds a single step of the reaction. This means no reaction will occur if the wells do not mix. Once the two wells are “slipped” into place, the reagents can mix for the reaction to occur. The slipping design enables simple operation of multiple steps with high throughput compartmentalization. A standard SlipChip can generate over 1000 uniform reaction compartments [100]. The SlipChip has been applied for number of targets including bacteria [103] and viruses [100]. It is also not limited to only PCR; the SlipChip has been shown to simplify LAMP reactions [104].

Flow cell loading and vacuum-assisted loading with oil digitization are some of the most common ways to load microwell arrays. Several new techniques have emerged for applications with PCR. Dispensing robots and automated loading processes reduce error and enable careful control of droplet volume. An automated capillary loading robot was developed for droplet 2D printing (Figure 3c) [98]. This technology used a capillary probe to print the PCR mix into a microdroplet array in a bed of oil on a hydrophobic silicon surface. This dispenser produced 100 2 µL droplets within 5 min and is easily programmable. Another dispensing robot was used for amplification-free detection of SARS-CoV-2 RNA to load 10^8^ femtoliter wells on a compact disk device [99]. To ensure highly efficient vacuum-assisted loading, a microfluidic device was designed using a pull-pull active digitization method for PCR. In this design, an alternative push and pull valve was used to fully load microwells to ensure complete loading. After the wells were fully loaded, the wells were sealed with an oil phase. The design offers ease of operation and high 99.5 ± 0.3% digitization efficiency for highly sensitive PCR [97].

#### 3.2.2. Droplet Microfluidics

Microfluidic droplet generation is the most common technique for compartmentalization of dPCR. After partitioning, the droplets are either stored on the device in a reaction chamber [105,106,107], transferred off the device and into another device or remain on the device under continuous flow [108,109,110]. Heating elements and integrated sample processing units can also be integrated in a microdevice.

Droplet microarrays have been applied for nucleic acid detection to eliminate complex microfabrication procedures. A microdroplet array using hydrophobic-hydrophilic patterning was used for isothermal amplification of human genomic DNA [111]. A commercial membrane was used to dispense micro-scale droplets onto a PDMS chip. The membrane pores allowed for the LAMP mixture to be dispensed onto the PDMS. Upon membrane liftoff, the remaining droplets were sealed with oil. This low-cost and simple design achieved a dynamic range of 11 to 1.1 × 10^5^ copies/μL for MS2 virus detection [112].

In addition to the common T-junction [113,114] and flow focusing [115,116,117] droplet generation, step emulsification (SE) has been also applied to PCR partitioning because of its simpler design and high throughput. In SE, droplets are formed by using a step design where one fluid stream flows through a shallow channel and breaks into droplets at a step because of the sudden change in interfacial tension. Compared to the T-junction and flow focusing, SE only needs to control the flow of the dispersed phase, and the droplet volume is determined by geometric design. A SE device demonstrated a sensitivity of 10 copies/μL with a dynamic range of approximately 4 logs for HER2 detection [118]. Another design used co-flow to stream sample and oil together and step emulsion to form the droplets. This device achieved a large dynamic range of 20 to 50,000 copies/μL [119]. In addition to using step emulsification by syringe pumping, a centrifugal step emulsification system was designed for RPA detection of L. monocytogenes. The step emulsification was conducted by centrifugal force, and droplet size could be controlled by adjusting the speed. More than 500 droplets per second per nozzle could be generated using this method [120]. Similarly, a centrifugal microfluidic device was combined with T-junction to generate droplets [121].

Several strategies have been presented to improve droplet generation and simplify the workflow in microfluidic devices. Some methods of droplet generation have emerged using simple pneumatic valves in microfluidics, such as in the “pushbutton-activated microfluidic dropenser” reported by Park et al. [122]. This method consists of a four-layer chip device containing a pneumatic layer with Tygon tubes, a membrane layer, a fluidic channel layer, and a cover layer. Oil and the PCR mixture are loaded into their corresponding channels, and droplets are formed at a cross channel by manually pushing the push button. Once the droplets form, they continue to flow through the channel through the first valve while the button is released, where they collect in the actuation chamber. When the button is pressed, the first valve closes, and the second valve opens, allowing the droplets to travel through the outlet and into a well plate for collection. This chip design allows for droplet generation and pumping within four channels simultaneously, and all channels are controlled with one push button [122].

To improve droplet generation throughput, a parallel microfluidic droplet generation system was reported [123]. In this design, eight flow-focusing droplet generators were integrated into a single microfluidic chip. Upon vacuum application, all eight generators simultaneously produced up to 20,000 droplets. After PCR cycling in the microfluidic chamber, the droplets were then passed through the detection unit in the downstream microchannel to count the result. Because of the high throughput generation, the device achieved single copy detection of human genomic DNA.

**Figure 4:**
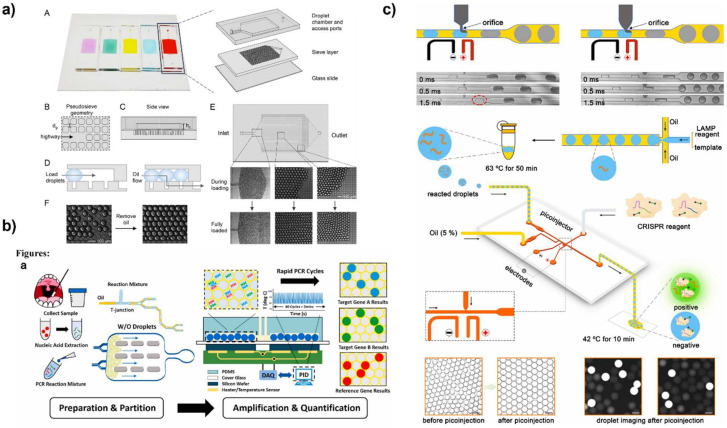
PDMS sieve trapping system for enhanced droplet loading. (**a**) Reprinted with permission from O’Keefe, C.; Kaushik, A.; Wang, T. Highly Efficient Real-Time Droplet Analysis Platform for High-Throughput Interrogation of DNA Sequences by Melt. Analytical Chemistry **2019**, *91* (17), Copyright 2019 American Chemical society [124]. (**b**) Multiplexed digital SARS-CoV-2 RNA detection using parallel droplet generation and color-coded fluorescence readout. Reprinted from Biosensors and Bioelectronics, Volume 188, Yin et al., “Ultrafast multiplexed detection system of SARS-CoV-2 RNA using a rapid droplet digital PCR system” p. 113282, Copyright 2021 with permission from Elsevier [126]. (**c**) Multistep digital LAMP and CRISPR microfluidic device for SARS-CoV-2 RNA detection. Reprinted from Biosensors and Bioelectronics, Volume 211, Wu et al., “DropCRISPR: A LAMP-Cas12a based digital method for ultrasensitive detection of nucleic acid” p. 114377, Copyright 2022 with permission from Elsevier [127].

For effective analysis of digital assays, it is important that all droplets be easily visible for imaging. Many microfluidic devices generate droplets, and the droplets are passed into a chamber or reservoir for incubation and readout. Within these chambers, the droplets will pack together using the space available. Often, this leaves dead volume and reduces the number of droplets that can be generated. To improve loading, a microfluidic platform was designed with a droplet trapping system to maximize space utilization. First, the droplets are generated using a flow focusing device and incubated in bulk using a thermal cycler. Then, they are loaded into the trapping system for readout (Figure 4a) [124]. The trapping system is a PDMS sieve layer that traps the droplets on the raised posts. Because of this design, the device exhibited 100% loading efficiency for up to 30,000 droplets. Single DNA molecules were partitioned to achieve a sensitivity of 0.8 copies/µL. Another microfluidic device employed a similar strategy to improve droplet packing, but instead using a sieve design, a honeycomb micropillar array were used for droplet trapping. Droplets were captured at a density of 160 to 250 droplets/mm^2^ and the assay achieved a sensitivity down to 10 copies/µL [125].

#### 3.2.3. Unconventional Compartmentalization Techniques for dPCR

Recently, there has been work in developing alternative droplet generation techniques for nucleic acid biosensing that does not require microfabricated devices. A microcentrifuge tube was modified with a droplet generation microchannel array to produce different-sized droplets by changing the centrifugal force. To form the droplets, the aqueous PCR mixture is loaded on top of the microchannel array. During centrifugation, the liquid passes through the hydrophobic coated array, and the aqueous stream is ejected through a nozzle and into the oil at the bottom of the tube to form a w/o emulsion. The device demonstrated excellent PCR dynamic range when compared with the commercially available Bio-Rad QX200 [128].

Inkjet printing has been shown as an alternative droplet generation system for PCR since inkjet technology is reliable for producing monodisperse droplets with CV < 5% [129]. Using the inkjet nozzles, the droplets were directly introduced into an oil continuous phase for downstream PCR. The device achieved HPV quantification with a dynamic range of over four orders of magnitude.

Acoustic streaming has also been demonstrated for compartmentalization. A vibrating capillary has been developed for droplet generation with a portable setup [130]. With this method, a generator, instead of pumps, provides the ability for stable droplet generation and tuning droplet size spanning two orders of magnitude. Since the dynamic range of dPCR experiments is highly dependent on the range droplet volumes, this design achieved a dynamic range of approximately six orders of magnitude, which is the widest to date. Additionally, the system is not restricted by device design or dimensions like in other microfluidic systems, so the number of generated droplets can be easily adjusted in real time.

### 3.3. Readout Methods for Nucleic Acid Detection

#### 3.3.1. Fluorescence

Fluorescence detection is the most widely used readout for dPCR as it is versatile and convenient for digital detection. Within the area of fluorescence detection, there are two common methods used: fluorescent dyes and fluorophore-labeled target-specific probes. In using a fluorescent dye such as SYBR Green, the dye molecules will intercalate with the double-stranded DNA and fluoresce upon excitation. Detection of the target can be monitored in reading time by an increase in the fluorescence intensity. Although these dyes are low-cost, they utilize non-specific binding to any double-strand DNA in the mixture, which can be an issue particularly if any source of contamination is present in the reaction vessel [131]. Alternatively, target-specific probes can be utilized. Like fluorescent dyes, these probes will not typically fluoresce until bound to the target sequence, although these sequences do not need a double strand [132]. Additionally, these fluorescent probes can be utilized in multiplexing. With multiplexing, each individual target would have its own probe sequence which can be modified with a target-specific fluorophore. Under different excitation and fluorescence conditions, the different targets can be detected [133].

Like in dELISA, the use of multiple fluorescent probes enables multiplexed detection in digital nucleic acid sensing. For example, a dLAMP assay was developed for detection of HCV and HIV by designing target specific fluorescent labeled primers that only activate when used in LAMP [134]. A real-time fluorescence curve recorded for the corresponding colors and quantified of multiple targets with high sensitivity of four copies/reaction. Similarly, for SARS-CoV-2 RNA detection, different fluorescence reporters were used in PCR to measure multiple genes (Figure 4b). This device not only was able to multiplex but is also measured samples in less than 5 min with high sensitivity at 10 copies/µL [126].

Fluorescence has also been mediated using CRISPR-Cas technology. With this method, a target-specific guide RNA strand forms a complex with a Cas nuclease. This complex then binds to the target nucleic acid sequence, and a reporter DNA strand is also introduced. Once the full complex is formed, the sequence cleaves the bond specifically at a protospacer-adjacent motif (PAM) adjacent to the binding site. This cleavage allows the fluorophore on the reporter DNA to fluoresce. The response from CRISPR readout is highly specific and produces strong signals, which further improves sensitivity [89,132,135]. CRISPR-based readout for nucleic acid detection can either be coupled with or without an amplification technique. For example, digital CRISPR has been coupled with LAMP and RPA and several microdevice platforms such a microfluidics [127,136] and microwell arrays [102,137,138]. Microwell arrays are advantageous for CRISPR readout because the isothermal amplification and CRISPR reaction mixes can be easily loaded into the microwell arrays for a rapid one-pot reaction [137,138]. In addition to using a one-pot design, the reactions can be translated to a SlipChip design for simple separation and merging of the amplification and CRISPR reaction steps [102]. By using amplification and CRISPR readout, these assays achieve sensitivities of 5 copies/µL [137] and 3 fM [127]. In an amplification-free CRISPR assay, the CRISPR assay mix and target sample were compartmentalized into water in oil droplets using a flow focusing microfluidic device. The isothermal reaction occurred over 60 min and showed a 50-fold improvement in sensitivity compared to other reported CRISPR assays. The high sensitivity of this assay was attributed to the careful optimization of the CRISPR reaction and digitation of the reaction [138]. Another group utilized a microwell array for amplification-free detection of SARS-CoV-2 RNA via CRISPR [91]. This assay achieved an LOD of ~10 fM in less than 5 min due and the assay was furthered by the same group by developing an automated dispensing robot to produce a large scale digital CRISPR array [99].

Combining multiple reaction steps using microfluidics has become incredibly valuable for nucleic acid biosensing. A two-step droplet generation system for LAMP + CRISPR readout was designed using a flow-focusing and T-junction device (Figure 4c) [127]. First, the amplification mix was prepared in droplets using the flow-focusing microfluidic. Then, the droplets were incubated and transferred to a second device, where they were mixed with the readout mix. This two-step device achieved an LOD of 3 fM within one hour for SARS-CoV-2 RNA detection. Another group used a combined cell lysis and isothermal amplification system to detect miRNA from single cells. Using a flow-focusing device, the cell lysis and hairpin amplification mix were merged with the cell sample to form whole assay droplets. The device measured 1 nM to 200 nM of miRNA within one hour and demonstrated a single-pot lysis and amplification assay [139].

#### 3.3.2. Colorimetric

Colorimetric detection is far less common that fluorescence; however, it offers advantages since results are readily visible without the need of an excitation source. For example, metal indicator hydroxynaphtol blue was used as an alternative to an intercalating fluorescent dye for LAMP [140]. Legionella species were detected visually on a digital microchip.

Many platforms for digital dPCR have been applied including microfluidics, microarrays, and microwell array. This field is expanding with more interest in isothermal amplification and amplification free techniques. Each platform and amplification method influences the device performance and operation.

**Table 2:** Summary of digital nucleic acid detection methods.

Target	Amplification Type	Platform	Generation Method	Readout	Limit of Detection	Range	Time	Reference
SARS-CoV-2 RNA	CRISPR	Microwell array	Flow cell	Fluorescence	5 fM	N/A	<5 min	[91]
SARS-CoV-2 RNA	CRISPR	Microwell Array	Manual loading	Fluorescence	1 GE/µL RNA	N/A	30 min	[90]
SARS-CoV-2 RNA	CRISPR	Microwell Array	Manual loading	Fluorescence	5 copies/μL	N/A	~1.5 h	[137]
HPV	LAMP	Microwell Array	Microfluidic loading	Fluorescence	N/A	N/A	1.5 h	[141]
pMD 18-T-HA β-actin DNA	LAMP	Microwell Array	Vacuum assisted loading and oil sealing	Fluorescence	N/A	N/A	1.5 h	[142]
*S. aureus* and *E. coli*	PCR	Microwell Array	Droplet Magnetofluidic Cartridge	Fluorescence	N/A	N/A	N/A	[143]
Chicken DNA	PCR	Microwell Array	Microfluidic loading	Fluorescence	N/A	N/A	30 min	[96]
*E. coli*	LAMP	Microwell Array	Microfluidic loading	Fluorescence	1 pg/μL	N/A	~1 h	[144]
VRE	LAMP	Microwell Array	Microfluidic loading	Fluorescence	11 copies	N/A	30 min	[145]
*S. agalactiae*, *P. mirabilis*, *S. aureus*, *K. pneumoniae*, and *E. coliare*	PCR	Microwell Array	Vacuum assisted loading and oil driven digitization	Fluorescence	N/A	10^4^ to 10^7^ CFU	4 h	[93]
λDNA	PCR	Microwell Array	Pressure actuation	Fluorescence	10 copies/µL	10 copies to 3600 copies/µL	N/A	[97]
SARS-CoV-2 RNA	PCR	Microwell Array	Dispensing robot	Fluorescence	3.9 copies/μL	8 aM–30 fM	10 min	[99]
BK Virus	PCR	SlipChip	Self-partitioning	Fluorescence	3.0 × 10^2^ copies/mL	3.0 × 10^4^ to 1.5 × 10^8^ copies/mL	N/A	[100]
SARS-CoV-2 RNA	LAMP/CRISPR	SlipChip	Self-partitioning	Fluorescence	4 × 10^2^ copies/mL.	N/A	1 h	[102]
SARS-CoV-2 RNA	LAMP	SlipChip	Self-partitioning	Fluorescence	344 to 901 copies/mL	2.74 to 4.81 log_10_	~30 min	[101]
HPV	LAMP	SlipChip	Self-partitioning	Fluorescence	N/A	N/A	~1 h	[146]
SARS-CoV-2 RNA	PCR	Microchamber array	Manual pipetting	Fluorescence	3.8 (N target region) and 3.0 (ORF1ab target region) copies per 20 μL	4 to 1000 copies	1.5 h	[147]
KRAS Gene	PCR	Microdroplet array	Manual loading	Fluorescence	N/A	3 to 3 × 10^3^ copies	5 min	[94]
miRNA-122	PCR	Microdroplet array	Capillary loading	Fluorescence	6 copies/droplet	3061 copies/cell to 79,998 copies/cell	N/A	[98]
λDNA	LAMP	Microdroplet array	Manual loading	Fluorescence	1 copy/μL	1 copy to 500 copies/μL	30 min	[148]
OLR1 gene	RCA	Microdroplet array	Hydrophobic hydrophilic patterning	Fluorescence	N/A	3 × 10^6^ to 3 × 10^3^ copies	30 min	[111]
Gram-positive and gram-negative bacteria	PCR	Microfluidic	Flow-Focusing	Fluorescence	10 CFU/mL	N/A	1 h	[149]
*E. coli*	PCR	Microfluidic	Wire guided droplet manipulation	Fluorescence	10^3^ genomic copies	5.2 × 10^5^−10^3^ genomic copies	15 min	[150]
Human Genomic DNA	PCR	Microfluidic	Flow Focusing	Fluorescence	N/A	Single copy to 100,000 copies	N/A	[123]
HBV	LAMP	Microfluidic	Flow Focusing	Fluorescence	N/A	10^1^ to 10^4^ copies	60 min	[151]
λDNA	PCR	Microfluidic	Flow Focusing	Fluorescence	Single copy	1.5, 0.5, 0.15 copy/droplet	N/A	[152]
*Porphyromonas gingivalis*, *Trepo-nema denticola* and *Tannerela forsythia*	PCR	Microfluidic	Flow Focusing	Fluorescence	125 CFU/µL	N/A	5 min	[153]
HER2	PCR	Microfluidic	Step emulsification	Fluorescence	10 copies/µL	4 log_10_	N/A	[118]
CDO1	PCR	Microfluidic	Flow-Focusing	Fluorescence	0.8 copies/µL	N/A	N/A	[124]
*E. coli*	PCR	Microfluidic	Flow-Focusing	Fluorescence	0.01 ng/µL	N/A	<1 h	[122]
λDNA	PCR	Microfluidic	Flow-Focusing	Fluorescence	N/A	4 copies/µL to 86.69 × 10^3^ copies/μL to 6.69 × 10^7^ copies/μL	N/A	[115]
PSA cDNA	PCR	Microfluidic	T-junction	Fluorescence	N/A	5 × 10^2^ to ∼5.5 × 10^4^ copies	<1 h	[113]
HPV	PCR	Benchtop reactor	Inkjet printing	Fluorescence	N/A	range 4 orders of mag	N/A	[129]
*E. coli*	PCR	Microfluidic	Co-flow	Fluorescence	N/A	1:10^5^ to 1:10^2^	~1 h	[118]
EpCAM cancer biomarker gene	PCR	Microfluidic	Flow-Focusing	Fluorescence	N/A	N/A	~1 h	[154]
16S *E. coli*	PCR	Microfluidic	Flow-Focusing	Fluorescence	1.56 nM	N/A	N/A	[155]
SARS-CoV-2 RNA	PCR	Microfluidic	Flow-Focusing	Fluorescence	10 copies/test	4 orders of magnitude	15 min	[156]
Bovine DNA	PCR	Microfluidic	Co-flow and step emulsification	Fluorescence	20 copies	20 to 50,000 copies/μL	15 min	[119]
SARS-CoV-2 RNA	PCR	Microfluidic	T-junction	Fluorescence	5 copies/test	10 copies to 1000 copies	<5 min	[126]
KRAS g12S	PCR	Microfluidic	Manual loading	Fluorescence	5 copies/test	5 copies per μL to 5 × 104 copies per μL	N/A	[92]
SARS-CoV-2 RNA	PCR	Microfluidic	Manual loading	Fluorescence	10 copies/µL	10 copies to 10,000 copies/µL	80 min	[95]
ACTB gene	PCR	Microfluidic	Flow focusing	Fluorescence	N/A	5000, 1500, 1000, 100, and 10 copies/μL	30 min	[125]
KRAS G12D	PCR	Microfluidic	Flow focusing	Fluorescence	N/A	N/A	N/A	[157]
circulating cell free DNA	PCR	Microfluidic	Flow focusing	Fluorescence	N/A	0.25 ng/mL to 11 ng/mL	N/A	[158]
PSA	PCR	Microfluidic	Dispenser	Fluorescence	0.48 ng/mL	0.5 to 30 ng/mL	~2 h	[159]
miRNA-21	PCR	Microfluidic	T junction	Fluorescence	N/A	N/A	<20 min	[114]
	PCR	Microfluidic	T junction	flow cytometry or gel	1 × 10^−7^	Five log_10_	N/A	[160]
*O. europaea*	PCR	Microfluidic	T junction	Fluorescence	10 nM	N/A	N/A	[161]
*E. coli* and *L. monocytogenes*	PCR	Microfluidic	Flow focusing	Fluorescence	10 CFU/mL	10 to 10^4^ CFU/mL	N/A	[105]
AOX gene	PCR	Microfluidic	Flow focusing	Fluorescence	N/A	N/A	13 min	[162]
HBV	PCR	Microfluidic	T junction	Fluorescence	N/A	N/A	N/A	[163]
ACTB gene	PCR	Microfluidic	Flow focusing	Fluorescence	N/A	90 to 9000 copies/µL	45 min	[106]
miRNA	PCR	Microfluidic	Flow focusing	Fluorescence	10 copies/µL	105 copies/μL to 10 copies/μL	<30 min	[107]
miRNA	PCR	Microfluidic	T junction	Fluorescence	N/A	300 to 3000 templates/µL	N/A	[110]
HIV	PCR	Microfluidic	Droplet printing	Fluorescence	10 copies/test	N/A	N/A	[164]
N/A	PCR	Microfluidic	Flow focusing	Fluorescence	N/A	N/A	N/A	[165]
PBMCs	PCR	Microfluidic	Flow focusing	Fluorescence	N/A	N/A	N/A	[166]
Salmonella	LAMP	Microfluidic	T junction	Fluorescence	3 fM	N/A	~1 h	[127]
Salmonella typhimurium	LAMP	Microfluidic	Flow focusing	Fluorescence	1 positive droplet per 250 CFU of *S. typhimurium*	N/A	30 min	[167]
Virus RNAs	LAMP	Microfluidic	Flow focusing	Fluorescence	4 copies	N/A	N/A	[134]
HIV	LAMP	Microfluidic	Flow focusing	Fluorescence	N/A	N/A	120 min	[168]
HBV	LAMP	Microfluidic	Flow focusing	Fluorescence	N/A	1 × 10^1^ to 1 × 10^4^ copies/μL	N/A	[169]
mRNA	LAMP	Microfluidic	Flow focusing	Fluorescence	N/A	N/A	N/A	[170]
*E. coli*, *E. faecalis*, and *Salmonella* Typhi	LAMP	Membrane	Peel off process	Fluorescence	N/A	11 to 1.1 × 10^5^ copies/μL	N/A	[112]
*Neisseria gonorrhoeae*	LAMP	Microfluidic	Flow Focusing	Fluorescence	~600 copies per μL.	N/A	N/A	[108]
*E. coli*	LAMP	Microfluidic	Emulsified by centrifugation	Fluorescence	N/A	15–1500 copies/μL	1.5 h	[171]
JAK2 V617F mutation	LAMP	Microfluidic	Centrifugal force	Fluorescence	N/A	10^1^ to 10^4^	1.5 h	[121]
HCT-116 genomic DNA	LAMP	Microfluidic	T junction	Fluorescence	5 copies/reaction	Five to 500,000 copies/reaction	1 h	[172]
vancomycin-resistant gene (vanA)	LAMP	Microfluidic	Flow Focusing	Fluorescence	1 copy/μL	50 to 2.5 × 10^3^ copies	~40 min	[173]
Artificial cells	LAMP	Microfluidic	Flow Focusing	Fluorescence	4 copies/droplet	4 to 8.7 × 10^9^ copies	N/A	[174]
HPV	RPA	Microfluidic	Flow Focusing	Fluorescence	1.1 copy/μL	6 orders of magnitude	10 min	[136]
HPV	RPA	Microfluidic	Flow focusing	Fluorescence	10 cp/μL	10 copies to 10,000 copies	30 min	[175]
miRNA	HCR	Microfluidic	Flow Focusing	Fluorescence	N/A	1 nM to 200 nM	~1 h	[139]
*L. monocytogenes*	RPA	Microfluidic	Centrifugal emulsion	Fluorescence	N/A	500 copies/μL to 4000	30 min	[120]
*Escherichia coli*, *Klebsiella pneumonia*, and *Proteus mirabilis*	FISH	Microfluidic	Lab disk	Fluorescence	~3 × 10^3^ bacteria/mL	Upper limit ~3 × 10^7^ bacteria/mL	1.5 h	[176]
miRNA	Auto-catalytic hairpin assembly	Microfluidic	Flow focusing	Fluorescence	0.34 pM	N/A	N/A	[177]
African swine fever virus, Epstein–Barr virus, and Hepatitis B virus	CRISPR	Microfluidic	Flow focusing	Fluorescence	0.5 pM	1750 to 17.5 copies/μL	1 h	[138]
*Shigella*, *Listeria monocytogenes*, *Bacillus subtilis*, and *Streptococcus pneumophila*	LAMP	Microfluidic	Manual generation	Fluorescence	<10 copies/μL	N/A	~1 h	[178]
miRNA 21	Circle strand displacement	Microfluidic	Flow focusing	Fluorescence	N/A	0.33–1.66 nmol/L	N/A	[109]
*Neisseria gonorrhoeae* 16S rRNA	RT PCR	Microfluidic	Flow focusing	Fluorescence	1aM	N/A	N/A	[179]
Legionella	LAMP	Microfluidic	Flow focusing	Colorimetric	100 fg/mL	100 fg/mL to 10^6^ fg/mL	N/A	[140]
*L. monocytogenes*	PCR	microcentrifuge tube	Centrifugal force	Fluorescence	N/A	Single copy to 2000 copy/µL	<1 h	[128]
HER2	PCR	Well	Vibrating Sharp tip capillary	Fluorescence	0.25 copies/µL	6 orders of magnitude	~1 h	[130]

Legend: Polymerase chain reaction (PCR), Loop-mediated isothermal amplification (LAMP), recombinase polymerase amplification (RPA) hybridization chain reaction (HCR), surface-enhanced Raman scattering (SERS).

## 4. Discussion

In this review, we summarize recent advancements in droplet technology for protein and nucleic acid, biosensing. Thanks to theoretical research and device engineering innovations, droplet bioassays have demonstrated their potential and value in healthcare, environmental, and public health settings. A range of innovations has been presented to improve droplet technology regarding droplet generation, manipulation, and assay sensitivity and performance. Each class of biomolecules has different technical requirements that must be considered when designing droplet assays. To date, several commercial instruments for digital bioassays are available on the market. For dELISA, the commercial system is based on the magnetic beads and microwell arrays (Quanterix Instrument). For digital PCR, droplet microfluidics (e.g., QX200, BioRad, Hercules, CA, USA) and microwell arrays (e.g., Quant Studio, ThermoFisher, Waltham, MA, USA; QIAcuity, Qiagen, Hilden, Germany) have been employed for commercial systems.

Most of the digital biosensing methods discussed here have yet reached the commercial market due to several reasons. First, some newly reported technologies still require time for optimization and maturation. Second, while many new digital sensing methods offer improvement over existing methods in terms of sensitivity and quantification, they are often associated with the increase of manufacturing complexity and system cost, and/or the decrease of system robustness and ease of use. To be competitive with existing commercial systems, the advantages offered by the new methods must outweigh the associated complication for commercialization.

One area that is especially promising for new digital sensing methods is POC testing as there is no existing commercial system for POC digital biosensing. Compared with conventional bulk assays, digital assay allows absolute quantification without calibration curves, improves detection sensitivity, and has better tolerances to the interferences in sample matrices. If these features can be transferred to POC testing, it will enable novel assays that address the key issues of existing methods in sensitivity and quantification. Currently, there are few examples of digital biosensing systems that are fully integrated for sample-to-answer analysis. Most efforts have been focusing on the utilization of simple compartmentalization methods and mobile imaging platform for signal readout. Little progress has been achieved for autonomous sample pretreatment and high-performance droplet generation, which are critical to the adoption and performance of POC digital biosensing. Biosensing from complex samples such as blood especially requires additional sample preparation such as cell lysis and extractions. Novel strategies that enable performing complex fluid and sample manipulation sequences in an automatic manner are urgently needed. In addition, streamlined data acquisition and analysis are also necessary to achieve true digital POC test.

Currently, digital biosensing remains primarily focused on sensing single targets. In today’s healthcare system, there is an increased need for measuring multiple targets in a single sample to provide a broader insight into disease states. Multiplexed biosensing assays give a clearer picture of valuable health information than single targets alone. Several examples of digital technology allow multiplexing of nucleic acids and proteins from a single sample; however, this research area is significantly smaller than that of studying new droplet-based technology [39,53,128,134].

In conclusion, droplet biosensing technology has flourished over the past decade because of bioassays and materials engineering advancements. Developing droplet assays is highly interdisciplinary and requires the integration of device engineering, biochemistry, and analytical chemistry. To produce high-performing biosensing systems, the current state of research in this field should be aimed at translating technology to real-world settings as a widely reliable technology.

## Data Availability

Not applicable.
